# Boys Don’t Cry? Rethinking Emotions and Manhood Through SEL in Pakistani Secondary Schools

**DOI:** 10.3390/bs16030458

**Published:** 2026-03-19

**Authors:** Rahat Shah, Sayed Attaullah Shah, Sadia Saeed

**Affiliations:** 1 Institute of Sociology, Goethe-Universität Frankfurt am Main, Westend Campus, Theodor-W.-Adorno-Platz 6, 60323 Frankfurt am Main, Germany; 2 Faculty of Social Sciences, Government College Peshawar, Faqir Abad, Shahi Bagh, Peshawar 25000, Pakistan; attaullahshah2018@gmail.com; 3 School of Sociology, Quaid-i-Azam University, Islamabad 45320, Pakistan; ssaeed@qau.edu.pk

**Keywords:** SEL, manhood, schoolboys, emotions, stigma, Pakistan

## Abstract

Global research on social–emotional learning (SEL) demonstrates robust benefits for student well-being and academic outcomes, yet SEL is still largely treated as gender and culturally neutral, with little attention to how it intersects with locally specific constructions of masculinity. We address this gap through a qualitative study in three urban secondary schools in Khyber Pakhtunkhwa, Pakistan, combining focus groups with boys aged 13–16 (*n* = 18), student interviews (*n* = 10), and teacher/counsellor interviews (*n* = 10). Using critical masculinity theory, the sociology of emotions, and transformative SEL, a reflexive thematic analysis identifies four patterns: (i) sadness and fear framed as status risks while anger signals strength, (ii) “switching off” feelings as masculinized emotion work tied to locally valued ideals of sabar (endurance) and izzat (honour), (iii) fragile “islands of care” where privacy and dignity enable conditional vulnerability, and (iv) SEL-like practices fostering empathy but also reinforcing stigma when emotions are labelled unmanly. We argue that SEL is a contested site where masculinities are reproduced and renegotiated, and we propose five findings-grounded design principles, including graduated emotional entry points, anti-ridicule norms, and indirect pedagogy for gender-attentive SEL that reduces stigma and supports non-violent masculinities in Pakistani secondary schooling.

## 1. Introduction

Pakistan is one of the world’s youngest countries, with recent national figures indicating that 67% of the population is under 30 (Government of Pakistan, 2025), and UNICEF estimates that over 107 million people are under 18 (UNICEF, 2025). For boys entering adolescence in this demographic landscape, everyday life is increasingly shaped by overlapping pressures, economic insecurity, exposure to violence, and limited access to mental health support. These burdens are complicated to address in settings where services are scarce and stigma is high. Pakistan shows this gap clearly, with just 0.19 psychiatrists per 100,000 people, mostly in major cities, and low overall investment in mental health (WHO, 2025a; Main Thompson & Saleem, 2025). Schools, where adolescents spend much of their daily lives, are expected to be protective institutions for well-being, belonging, and social development, yet, in many Pakistani settings, schooling is also a site where violence and emotional regulation are shaped through discipline and peer hierarchies (Khan et al., 2025; WHO, 2025b). Empirical work documents widespread exposure to harsh punishment; a recent multi-site analysis shows that over 90% of boys reported corporal punishment at school (Khan et al., 2025), alongside high levels of bullying and peer victimization linked to emotional and behavioural difficulties (Naveed et al., 2020; Siddiqui & Schultze-Krumbholz, 2023).

Within these school ecologies, boys’ emotional lives are regulated by culturally resonant expectations of manhood, and everyday injunctions such as *boys don’t cry* or *be a man* are not merely sayings; they function as social rules that mark sadness, fear, and vulnerability as threats to masculine status while normalizing emotional toughness and, often, anger. A growing body of Pakistani research links masculine norms to reduced help-seeking and greater stigma around emotional disclosure (Shah & Shah, 2024; de Visser et al., 2022), alongside qualitative evidence that adolescent boys suppress emotions due to fear of ridicule and cultural expectations of masculinity (Ahmmed & Khan, 2024). In parallel, Pakistan faces serious challenges around self-harm and suicide that remain under-discussed because of stigma and gaps in official reporting, as WHO-estimate-based analyses suggest male suicidal deaths substantially exceed female deaths, while self-harm is likely much more common than recorded suicides (Asad et al., 2022). Newspaper-based surveillance further indicates recurring adolescent suicide cases, with boys comprising a slight majority, findings that underscore both urgency and undercounting (Imran et al., 2023).

At the same time, global evidence on social–emotional learning (SEL) shows robust benefits for student well-being, relationships, behaviour, and academic outcomes across diverse schooling contexts (Cipriano et al., 2023, 2024). International guidance increasingly positions SEL as central to education reform and to children’s psychosocial well-being, including in crisis-affected and low-resource settings (UNESCO, 2024; UNHCR, 2024). Yet much SEL research and programming still operates as if emotions are gender-neutral skills, insufficiently theorizing how SEL competencies are learned within unequal gender orders, or how SEL activities can unintentionally reproduce stereotypes about what boys and girls *should* feel and show. Scholarship on emotional pedagogy demonstrates that classrooms are deeply gendered spaces where emotional expression is encouraged, sanctioned, or ridiculed in patterned ways (Evans, 2017), and equity-oriented approaches such as transformative SEL argue that identity, power, and social norms must be explicit rather than treated as background context (Jagers et al., 2025). Despite these advances, South Asian school contexts, and Pakistan in particular, remain under-represented in masculinity-informed SEL scholarship, and we know too little about how schoolboys themselves narrate emotion, discipline, peer status, and *being a man* in relation to SEL-like practices already present in schools.

Against this backdrop, and drawing on qualitative data from three urban secondary schools in Khyber Pakhtunkhwa province of Pakistan (two government, one private), we ask: How do schoolboys in Pakistani secondary schools talk about anger, sadness, fear, and vulnerability, and in what ways do existing or potential SEL practices open up or close down space for non-violent, emotionally expressive masculinities?

### 1.1. Masculinity and the Costs of Emotional Silence

Critical masculinity scholarship has long emphasized that masculinities are not fixed traits but socially produced patterns of practice that are rewarded, policed, and contested within institutions such as schools. The concept of hegemonic masculinity foregrounds how certain ideals of manhood become culturally dominant, often privileging toughness, emotional restraint, and authority, while marginalizing alternative masculinities (R. W. Connell & Messerschmidt, 2005). In many adolescent peer cultures, masculine status is maintained through the management of vulnerability, where boys learn that visible sadness, fear, or need can be interpreted as weakness, while emotional control signals strength. This does not mean boys feel less; rather, they often learn to translate distress into more socially acceptable displays such as anger, sarcasm, risk-taking, or withdrawal.

Empirical work in Pakistan aligns with broader masculinity-and-emotion scholarship while clarifying locally specific mechanisms through which boys learn emotional restraint. In a comparative study of young men in Pakistan and the United Kingdom, stronger endorsement of traditional masculinity beliefs, particularly in Pakistan’s more strongly patriarchal gender context, was associated with lower willingness to seek help for both physical and mental health concerns (de Visser et al., 2022). Further studies document how peer ridicule, honour-inflected expectations, and authoritarian family socialization make emotional expression feel socially risky, normalizing suppression as a protective strategy and framing vulnerability as a threat to masculine standing (Ahmmed & Khan, 2024; Shah & Shah, 2024). Read through critical masculinity theory, these findings reflect how hegemonic ideals are sustained not only by what boys believe, but by what they anticipate others will reward or punish, especially in peer cultures where status is continuously negotiated (R. W. Connell & Messerschmidt, 2005).

These sociological observations are corroborated by research in developmental affective science, where studies on emotion socialization demonstrate that from early childhood, boys receive less encouragement than girls to express internalizing emotions such as sadness and fear, while externalizing expressions such as anger are more frequently tolerated or even reinforced for boys (Chaplin & Aldao, 2013). Similarly, it is argued that gender differences in emotional expression are best understood not as innate traits but as products of socialization, cultural display rules, and relational contexts, a position consistent with our theoretical framing through Hochschild’s feeling rules (Brody & Hall, 2008). Developmental research further shows that male adolescents tend to employ more emotional suppression and avoidance strategies compared to female adolescents, who report greater use of social support-seeking and reflective strategies (Zimmermann & Iwanski, 2014), and that these gendered regulatory patterns become more pronounced during adolescence as peer conformity pressures intensify. This affective science evidence underscores that the emotional silence documented among boys in our study is not idiosyncratic but reflects broader, well-documented developmental patterns shaped by the interaction of gender socialization and peer ecology.

These patterns matter beyond individual well-being because they shape the moral and emotional climate of schooling, where vulnerability is stigmatized, aggression and dominance can become viable routes to recognition, particularly in school environments marked by peer victimization and punitive discipline (Siddiqui et al., 2025). Alongside the school violence and bullying patterns documented above (Khan et al., 2025; Naveed et al., 2020; Siddiqui & Schultze-Krumbholz, 2023), discipline itself becomes a site where emotional regulation is learned through fear and reputational management. Importantly, masculinity norms do not operate in a vacuum when economic insecurity, family pressures, and exposure to violence are common; expectations that boys must remain unshakeable can intensify isolation, delay help-seeking, and make emotional silence feel like the only socially *safe* option, despite its longer-term psychosocial costs (de Visser et al., 2022; Hochschild, 1983).

### 1.2. Discipline, Peer Hierarchies, and the Gendered Regulation of Emotion in Pakistani Schools

Like everywhere, schools in Pakistan are key sites where these gendered emotional dynamics are institutionally embedded, and as documented above, the combination of widespread corporal punishment, peer victimization, and punitive disciplinary cultures creates school environments where emotional expression is especially risky; being seen as soft invites ridicule from peers, while showing distress can be met with punishment rather than care (Khan et al., 2025). In such conditions, boys learn to pre-empt vulnerability, stay hard, and manage threats to status through aggression or emotional numbing (Naveed et al., 2020).

Even when teachers recognize distress, they may lack training, time, or institutional backing to respond in non-stigmatizing ways (Imran et al., 2023). This matters because schools are not merely educational spaces; they can function as front-line institutions for early identification of psychosocial difficulties, norm change, and prevention. When this potential is unrealized, boys’ distress may circulate through the very channels that schools already know how to manage, discipline, exclude, or moralize about *being a man* (Connors et al., 2022).

Crucially, classroom emotion is not just individually experienced, it is socially organized, and research on emotional pedagogy shows that schools routinely teach feeling rules, implicitly and explicitly, and that these rules are gendered: different emotional displays are encouraged or sanctioned for boys and girls, shaping distinct emotional subjectivities (Evans, 2017). In Pakistani contexts where masculine respectability is tightly tied to emotional control, this gendering can be intensified, and the same SEL activity (e.g., naming feelings, discussing worry, reflecting on mistakes) may be interpreted as character-building for some students but as status-threatening for others, especially when peer cultures equate openness with weakness (Shah et al., 2022; Shah & Shah, 2024).

These dynamics are intensified in the Khyber Pakhtunkhwa context of this study, where Pashtun masculinity is organized through Pashtunwali, a moral code that foregrounds honour (nang), courage (meṛāna), and retributive justice (badal) as defining virtues of manhood (Ahmed, 2013; Lindholm, 1982). Within this framework, emotional endurance is not a personal trait but a socially demanded performance tied to family reputation (izzat), where visible vulnerability reflects not only on the individual boy but on his household’s standing, and Pashtun boys learn from early childhood that toughness and stoicism must be actively displayed in public settings including schools (Ahmed, 2013; Shah & Shah, 2024). The emotional silence described by our participants is therefore not adequately captured by generic “masculinity norms” alone, it is shaped by a locally specific honour economy in which emotions carry reputational costs beyond the individual. This specificity also sharpens the critique of SEL, which is not only gender-neutral but culturally neutral, designed largely in Western contexts and rarely engaging with how emotion talk is regulated by honour-based relational systems or the dynamics of post-colonial educational institutions in the Global South (McCoy & Hanno, 2023; Cipollone et al., 2022).

### 1.3. SEL’s Promise and Why Gender Matters

Building on the robust meta-analytic evidence for SEL’s benefits discussed above (Cipriano et al., 2023, 2024), global policy increasingly positions SEL as relevant beyond high-income contexts, including in low-resource and crisis-affected settings (UNESCO, 2024; UNHCR, 2024). In Pakistan and neighbouring contexts, *life skills* initiatives (often overlapping with SEL) have been promoted as vehicles for adolescent well-being and empowerment, suggesting both policy traction and practical entry points for SEL-like work (Farooq & Alyana, 2025).

However, SEL’s expansion also raises conceptual risks when it is treated as a culturally and gender-neutral toolkit. Equity-oriented frameworks, such as transformative SEL, argue that SEL should explicitly engage with identity, belonging, power, and social norms rather than assuming a one-size-fits-all model of *healthy* emotion (Jagers et al., 2025). Related critiques show how SEL can carry a hidden curriculum, in which teaching *appropriate* emotions and behaviours may align students with dominant norms unless reflection on power and inequality is built into the pedagogy (Cipollone et al., 2022). For boys in particular, SEL activities that invite emotional disclosure may become arenas of masculine policing unless classrooms also address the social penalties attached to vulnerability.

What remains underdeveloped, especially in Pakistan, is empirically grounded, school-based research that examines how boys narrate anger, sadness, fear, and vulnerability inside school life, and how SEL-like practices interact with local masculinity norms, either reducing stigma or inadvertently reproducing it (Shah, 2026). This article addresses that gap through a qualitative, masculinity-informed analysis of SEL as a contested terrain where emotions and manhood are simultaneously reproduced and renegotiated.

### 1.4. Theoretical Framework

This study is guided by critical masculinity studies, which conceptualize masculinities not as individual traits but as socially produced, relational, and hierarchical configurations of practice that are made and remade in institutions such as schools (R. W. Connell & Messerschmidt, 2005). We use hegemonic masculinity as a sensitizing concept to examine how particular ideals of *real manhood* become culturally dominant among boys (e.g., toughness, emotional control, willingness to retaliate), while other ways of being a boy (e.g., expressing fear, sadness, tenderness) are subordinated, ridiculed, or coded as weak. This perspective helps us treat everyday phrases like “boys don’t cry” and *“mard ban, or be a man”* not as mere attitudes but as normative rules that shape peer status, discipline, and moral worth in school life (R. W. Connell & Messerschmidt, 2005).

At the same time, we approach hegemonic masculinity as a sensitizing rather than deterministic concept. In Pashtun-majority Khyber Pakhtunkhwa, emotional restraint is not solely a product of peer policing; it is also embedded in locally valued moral concepts such as *sabar* (patience/endurance) and *ghairat*/*izzat* (honour/respect) that frame stoicism as a positive virtue rather than a pathology (Ahmed, 2013). We therefore adopt a dual analytic lens, with critical masculinity theory to examine when emotional restraint functions as hierarchical control, while also remaining attentive to participants’ own moral vocabularies and to the possibility that some forms of regulation serve protective, culturally valued purposes that cannot be reduced to deficit (de Visser et al., 2022; Wojnicka & de Boise, 2025).

To locate these masculine norms in school practice, we also draw on Connell’s notion of gender regimes, the idea that organizations have patterned gender relations that structure power, labour/roles, emotional relations, and symbolic meanings (R. W. Connell, 2002; R. Connell, 2005). In this view, Pakistani secondary schools are not neutral backdrops where gender simply *plays out*; rather, they are active sites of gender-making, through discipline, classroom interaction, peer hierarchies, and the informal cultures of corridors and playgrounds. Using the gender-regime lens directs attention to how rules about masculinity are embedded in institutional routines (e.g., punishment, public shaming, *hardening* boys) and how these routines condition which emotions can be shown safely, by whom, and in what situations (R. W. Connell, 2002; R. Connell, 2005).

Because the study is centrally concerned with emotions, it is further grounded in the sociology of emotions, especially Hochschild’s concepts of feeling rules and emotion work (Hochschild, 1979, 1983). Feeling rules specify what people should feel and display in particular contexts (e.g., *a boy should not cry*, *anger is acceptable for boys*), while emotion work captures the efforts individuals make to align inner experience and outward expression with those rules (Hochschild, 1979, 1983). We link this to emotional pedagogy, which highlights that schooling teaches emotions through micro-practices, such as praise, reprimands, jokes, silences, and the gendered policing of who may express vulnerability (Evans, 2017). Together, these lenses allow us to analyze boys’ narratives as evidence of how emotions are governed and learned within a gendered school ecology.

At the same time, we avoid treating masculinity as only domination, and to theorize possibilities for change, we incorporate the concept of caring masculinities, which describes emerging masculine identities and practices that reject domination and embrace care, relationality, and emotional openness (Elliott, 2016). Caring masculinities provides a language for identifying and interpreting *islands of care* inside schools, moments where teachers or peers legitimate worry, failure, family stress, or tenderness without immediate status loss. We also remain attentive to recent debates that caution against romanticizing *care* and call for specifying when, for whom, and under what conditions caring masculinities become socially viable (Wojnicka & de Boise, 2025), which helps us treat alternative masculinities as contested and uneven rather than as a simple moral counter-model.

Finally, the study is anchored in SEL theory as both a skills framework and a normative educational project, and we use the CASEL framework’s five competency domains, self-awareness, self-management, social awareness, relationship skills, and responsible decision-making, as a practical map of the kinds of emotional and relational capacities that schools attempt (explicitly or implicitly) to cultivate (CASEL, 2020). However, we interpret SEL through the lens of transformative SEL, which argues that SEL must engage questions of identity, power, belonging, and inequality rather than being treated as a culturally or gender-neutral technique (Jagers et al., 2025). This is pivotal for our argument that SEL is a contested terrain in which boys’ emotions and masculinities are simultaneously reproduced and renegotiated: depending on how SEL-like practices are framed and enacted, they may either (a) reinforce gendered feeling rules that label vulnerability as *unmanly*, or (b) expand the school’s emotional repertoire and make space for non-violent, emotionally expressive masculinities (CASEL, 2020; Jagers et al., 2025).

In combination, these theories guide our analysis in three ways, (1) they direct attention to boys’ emotion-talk as situated performances within hegemonic masculine hierarchies (R. W. Connell & Messerschmidt, 2005); (2) they frame schools as gendered institutions that teach feeling rules through everyday pedagogic and disciplinary practices (R. W. Connell, 2002; Evans, 2017; Hochschild, 1979); and (3) they provide conceptual tools to evaluate when SEL practices function as mechanisms of norm reproduction versus norm transformation, including the emergence (and limits) of caring masculinities in school life (Elliott, 2016; Jagers et al., 2025; Wojnicka & de Boise, 2025).

## 2. Materials and Methods

### 2.1. Study Design and Approach

This article reports findings from a qualitative, multi-site school study conducted in three urban secondary schools (two government schools and one private school). The study was designed to explore how adolescent boys describe and make sense of emotions (especially anger, sadness, fear, and vulnerability) within the moral and interactional worlds of school life, and how existing or potential SEL-like practices may, intentionally or unintentionally, shape the emotional *rules* attached to manhood. Given the study’s focus on meaning-making, norms, and socially regulated emotion talk, a qualitative design was most appropriate for capturing boys’ lived accounts in context and for attending to ambivalence, contradiction, and silence around stigmatized feelings (Patton, 2015; Tracy, 2010), and reporting is informed by established qualitative reporting guidance (Tong et al., 2007).

We selected schools in Khyber Pakhtunkhwa (KP) purposively for three reasons. First, as a Pashtun-majority province, KP provides a context where masculine norms are shaped by the culturally specific honour code of Pashtunwali, which foregrounds emotional endurance, courage, and stoic composure as core masculine virtues (Ahmed, 2013; Shah & Shah, 2024), making it a theoretically productive setting for examining the intersection of masculinity and emotional expression. Second, KP’s education system includes both government and private schools with markedly different disciplinary cultures and resources, enabling institutional comparison, and third, KP has been the site of recent educational reform efforts, including life-skills and psychosocial support initiatives, yet remains under-represented in SEL and masculinity scholarship compared to Punjab or Sindh provinces.

### 2.2. Sampling and Participants

We used purposive sampling to select school sites and participants to capture institutional variation relevant to emotional socialization and school discipline, particularly differences between public and private schools in urban areas. Within each school, we recruited adolescent boys who were enrolled in lower-to-mid secondary grades and who fell within the target age group, 13–16 years. Participation was voluntary, and in total, data were generated through three focus group discussions (FGDs) with boys (*n* = 18) across the three FGDs, semi-structured individual interviews with boys (*n* = 10), and semi-structured interviews with educators, including teachers and school counsellors (*n* = 10).

In total, the study generated data through 23 distinct data-collection events (3 FGDs and 20 semi-structured interviews) with 38 participants across three school sites. Empirical reviews of saturation in qualitative research indicate that theme saturation is typically reached within 9–17 interviews and 4–8 focus groups in studies with homogeneous populations and narrowly defined objectives, and our dataset falls within these empirically established ranges (Hennink & Kaiser, 2022; Guest et al., 2006). Importantly, in reflexive thematic analysis, sample adequacy is evaluated not through a procedural saturation checkpoint but through the researcher’s capacity to generate rich, coherent, and contextually grounded patterns of meaning (Braun & Clarke, 2021). The study’s exploratory and theory-informed design aimed to produce information-dense, contextually embedded accounts of how boys narrate emotion within school masculinity regimes, rather than to achieve population-level generalizability, yet we recognize the scope of our claims and address the boundaries of transferability in the discussion’s limitations section.

The researchers had no prior professional or personal relationships with the participating schools, teachers, or students, and access was negotiated through formal institutional channels. The first author contacted school principals via written requests explaining the study’s aims, and access was granted after meetings with school heads and, in the case of government schools, district education officials. No school was selected based on prior research involvement or personal connections.

The study was approved by the Ethics Committee, School of Sociology, Quaid-i-Azam University, Islamabad (Protocol No. QAU/SOC/2025-9). Ethical procedures were followed in line with widely used guidance for research with human participants, with particular attention to informed participation, confidentiality, and minimizing risk when discussing sensitive topics such as distress, conflict, and self-harm (World Medical Association, 2013), and for minor participants, appropriate assent and guardian consent procedures were used, and participation/non-participation had no consequences for students’ standing at school.

### 2.3. Data Collection

Data collection combined FGDs and semi-structured interviews to capture both collective norm talk and individual emotional narratives. FGDs were used to examine how boys co-produce meanings of *strength*, *weakness*, *anger*, *crying*, *fear*, and *vulnerability*, including how they describe peer teasing, masculine status, and disciplinary expectations. Focus groups are well-suited to exploring normative topics because participants respond to one another’s accounts, thereby revealing shared language, disagreements, and the boundaries of what can be said safely (Morgan, 1997). Individual interviews with boys created additional space for discussing sensitive experiences, such as family stress, humiliation, fear, sadness, or moments of emotional overwhelm, that might be difficult to disclose in front of peers.

All focus group discussions and interviews were conducted in Pashto and Urdu (depending on participants’ preference), as these are the primary languages of communication in KP schools. The first and second authors, both native Pashto speakers with fluency in both languages, conducted all data collection without the use of external interpreters. FGDs lasted approximately 45–60 min each and were conducted in private classroom spaces arranged by school administration during non-instructional periods. Individual interviews lasted approximately 25–40 min and were held in private rooms (counselling offices or empty classrooms) to ensure confidentiality, and all sessions were audio-recorded with participants’ (and, for minors, guardians’) informed consent, and recordings were transcribed verbatim in the original language by the second author before being translated into English for analysis by the first author. Translation accuracy was cross-checked through back-translation of selected extracts by a bilingual colleague not involved in data collection.

Semi-structured interviews with teachers and school counsellors focused on how educators interpret boys’ emotional displays (e.g., anger outbursts, withdrawal, crying), what responses are considered legitimate or effective, and which school routines resemble SEL (e.g., pastoral talks, informal mentoring, circle discussions, group projects) and how these are framed. Across all participant groups, interview/FGD guides included open-ended prompts on (1) situations that trigger anger, sadness, fear, or shame at school and at home; (2) how boys learn what *real men* should feel and show (3) peer and teacher reactions to vulnerability, and (4) experiences with, and perceptions of, relationship- and emotion-focused practices in school life that align with SEL competencies (CASEL, 2020). Consistent with ethical guidance for sensitive topics, we avoided pressuring participants to disclose personal or identifying details and instead, prompts were phrased to allow discussion in general terms (*boys in this school*…) as well as personal narratives if participants chose to share them (World Medical Association, 2013).

Regarding positionality, the first author (male, Pashtun, based at a German university) has prior research experience on masculinities and youth violence in KP, while the second author (male, Pashtun, early-career researcher and lecturer, based in Pakistan) conducted all fieldwork and established an informal rapport with adolescent participants as a younger male from the same ethno-linguistic community. Both authors were mindful that their gender and institutional positioning may have influenced what boys felt safe sharing, and we addressed this through iterative reflexive memoing, team debriefing, and triangulation with teacher/counsellor accounts. The third author (female, senior academic) provided critical oversight, challenging interpretive assumptions during analysis.

### 2.4. Data Analysis

We analyzed the data using reflexive thematic analysis, which is a flexible approach for identifying patterned meaning across qualitative datasets while attending to context, power, and researcher positionality (Braun & Clarke, 2006; Braun & Clarke, 2019). Analysis proceeded iteratively through (1) familiarization with the dataset, (2) generating initial codes, (3) developing candidate patterns of shared meaning, (4) reviewing and refining these patterns in relation to the coded extracts and the whole dataset, and (5) defining and writing analytically coherent accounts (Braun & Clarke, 2006).

The analysis was informed by sensitizing theoretical concepts from critical masculinity studies (especially hegemonic and caring masculinities) and by SEL competency frameworks (CASEL, 2020; R. W. Connell & Messerschmidt, 2005; Elliott, 2016). Practically, this meant we combined inductive attention to participants’ own terms and narratives with theory-informed interpretation of how emotional expression is policed, rewarded, or made risky in school settings, and how SEL-like practices may widen or narrow boys’ emotional repertoires. To strengthen the credibility and transparency of interpretation, we used regular analytic memoing and team discussion to check whether claims were grounded in multiple instances across sites and participant groups, and we actively attended to discrepant and less typical accounts rather than treating them as noise (Nowell et al., 2017; Tracy, 2010). While such accounts were actively sought during coding and interpretation, they emerged only as isolated exceptions rather than as a recurring pattern across participants. This absence of strong empirical deviance was itself analytically significant, indicating the depth and durability of hegemonic masculine expectations in the studied settings, even where boys’ narratives revealed ambiguity, tension, or subtle negotiation. The results section that follows, therefore, foregrounds the dominant patterns across participants’ accounts, while also noting moments of ambiguity, tension, and limited variation where these help clarify how hegemonic masculine norms were reproduced and negotiated in the studied settings.

## 3. Results

Across the three schools, boys’ accounts showed that emotions were not simply private experiences, but status-laden performances organized through peer surveillance and institutional discipline. Read through the sociology of emotions, these accounts also reveal gendered feeling rules, shared norms about which emotions are legitimate for boys to show, and the everyday *emotion work* boys perform to remain recognizable as properly masculine (Hochschild, 1979, 1983). Finally, SEL-like practices surfaced as ambivalent arenas, and they could expand boys’ emotional repertoires under certain conditions, but could also reproduce stigma if vulnerability was framed as unmanly or left unprotected from ridicule (Evans, 2017; Jagers et al., 2025).

### 3.1. Emotions as Weakness and Risk

Boys described sadness, fear, and crying as public liabilities that could quickly become reputational damage, and emotional talk repeatedly returned to the idea that vulnerability is *dangerous* because it invites mockery, challenges status, and signals incompetence in masculine terms. Anger, by contrast, was narrated as socially intelligible and sometimes strategically useful, an emotion that could deter harassment or reassert respect. These accounts illustrate how hegemonic masculinity operates less as an abstract ideal and more as a peer-enforced rule system shaping what boys can safely display (Way, 2011) and also showing how *feeling rules* designate some emotions as shameful for boys, producing an emotional hierarchy in which anger becomes a culturally sanctioned translation of distress (Brody & Hall, 2008) A government school student, named Bilal, narrated his views in one of the FGDs in this regard, arguing:

*“If a boy cries in class, he’s labelled immediately ‘kamzor (weak) ‘soft,’ or they say ‘ta jenotarry ye (you are like a girl)’. The teasing doesn’t stop… it becomes a joke for weeks… And it stays with him.”*

Bilal frames crying as a long-term stigma marker, something that becomes *sticky* to identity rather than a momentary event. This echoes the social logic of hegemonic masculinity, where vulnerability threatens one’s position in a gendered status order (R. W. Connell & Messerschmidt, 2005). The phrase *it stays with him* highlights that emotional expression is treated as evidence of character failure rather than *as* a situational response. The quote also illustrates gender policing through feminization, where vulnerability is sanctioned by equating it with being *like a girl*, making masculinity something maintained through ridicule and reputational control.

Another participant shared his views, saying:

*“You are scared of teachers who beat or humiliate you, and also of other boys. But you can’t admit that fear… and if you do, they say you are a coward, you can’t handle the things…and then it becomes a reason for bullying.”*

(Usman, 14, Private School C, Interview)

Usman’s account links fear to a materially unsafe school environment, where the threat of being beaten or victimized is real, but the emotional response must be managed to avoid further risk. This shows how fear becomes socially illegible for boys, where displaying it is read as incompetence (*can’t handle things*) and weakness, which then triggers peer sanction through bullying. In this way, masculine *emotional control* functions less as a personal trait than as a protective strategy under peer surveillance, where hiding fear is part of staying safe and maintaining status.

*“Anger is allowed when someone insults your family or dignity… If you stay quiet, they think you are weak. Showing anger makes others stop and take you seriously.”*

(Hamza, 16, Government School B, Interview)

Hamza frames anger as a socially legitimate emotion that functions as a status-protecting performance, unlike fear or sadness, which risks humiliation, and his account is that anger interrupts teasing, signals toughness, and compels recognition (*they take you seriously*), thereby reshaping peer interactions through deterrence. This suggests that in contexts where vulnerability is stigmatized, anger can become a permitted channel for distress and self-defense, reinforcing a school masculinity where respect is secured through emotional hardness rather than openness (Schrock & Schwalbe, 2009).

One of the teachers contrasted peer reactions to crying versus anger, showing how schools can become training grounds for gendered emotional norms. He argued:

*“If a boy is crying, students mock him and remember it for a long time… They call him weak…but showing anger in a controlled way is accepted. Also boys who shout or stand their ground are seen as strong…”*

(Teacher, Government School, Interview)

This indicates how these *rules* are socially produced through peer response and not only family socialization, strengthening the argument that schools are central to the gendering of emotion and masculine identity formation. Another teacher also presented similar views, arguing:

*“Boys tell each other, ‘Don’t behave like a girl,’ when someone looks upset…and these sanctions control each other.”*

(Teacher 2, Private School C, Interview)

This reveals the gendered mechanism of policing, where emotional regulation is enacted through feminization and ridicule. The phrase *control each other* highlights masculinity as a collective project maintained through sanctions, aligning with hegemonic masculinity’s relational and hierarchical character (Pascoe, 2011), and how SEL tasks that invite emotion talk may activate gendered stigma unless carefully scaffolded (Jagers et al., 2025).

### 3.2. Learning to Switch Off

Beyond immediate stigma, boys described a longer socialization trajectory, where they learn to *switch off* feelings, keep a neutral face, and avoid emotional language. In the Khyber Pakhtunkhwa context, where masculinized respectability is often tied to being *sakht* (tough) and *mardana* (manly) (Shah & Shah, 2024), emotional restraint was narrated less as a personal preference than as a practical survival skill, where they described monitoring their bodies (face, voice, tears) and pre-empting vulnerability before others can weaponize it. Yet the same accounts also point to the costs of this strategy, and these boys linked emotional numbing to feeling alone, becoming easily irritated, and losing interest in schoolwork, suggesting that *becoming mard* (man) through endurance can also mean carrying distress without relational outlets. This was evident from interview accounts, as a student named Saad argued:

*“You do not need to cry in front of people… controlling yourself is a skill, too. Relax by drinking a glass of water, so you won’t need to cry or express your sadness, you know… (.) Things return to normal because there is no safe way to express sorrow…”*

(Saad, 16, Government School B)

Saad’s wording implies deliberate self-regulation and an internal instruction to reduce emotional intensity, which is a form of emotion work in Hochschild’s sense: managing feelings to meet social expectations (Hochschild, 1979). The goal is survival (*drinking a glass of water to relax),* suggesting that patience is a coping mechanism in risky environments, yet may also reduce reflective processing. Another student presented his views and experiences in this regard, arguing:

*“Even when the stick hurts so much…I try to control my emotions and never try to show my class fellows that I am being hurt because they will make jokes and start making fun of me.”*

(Boy, 14, Government School A)

This highlights the embodied dimension of emotion work, where control of the face and visible affect occurs under peer surveillance, showing how minor signs of distress can escalate into public attention, making *normality* a protective performance rather than a neutral state.

Our data further show that, although being silent in stressful situations is viewed as a weakness, it is also associated with psychological consequences. As argued by a participant:

*“I think no one knows what stress and anxiety are, and no one talks about it. People think you are weak… if you say so…so you keep quiet. But if you are stressed and don’t do anything about it… Then inside, it feels heavy, and you get angry quickly…”*

(Boy, 15, Private School C, FGD)

These accounts show how *keeping quiet* operates as gendered emotion work where boys learn that naming stress is socially legible as weakness, so silence becomes a practical strategy for protecting masculine status in front of peers and adults. Yet the quote also exposes the cost of this strategy, where unspoken anxiety accumulates as an embodied *heaviness* that is displaced into irritability and quick anger, one of the few emotions still permitted as masculine. In this way, switching off does not remove distress; it reshapes it into harder, more socially acceptable affective expressions that can strain relationships and undermine engagement with learning. One of the teachers narrated this further in his interview account:

*“Some boys become almost blank in class… like you know… not having any facial expression, no reaction, as if nothing touches them anymore. Even when something goes wrong, some of them will just sit there quietly… and act like it doesn’t matter. But then … sometimes, that same person in the corridor or during a game may suddenly explode over very small things… like someone bumps them, a joke is made, and they lose control all of a sudden…”*

(Teacher 3, Government School B, Interview)

This shows a pattern consistent with emotional numbing followed by sudden anger, where withdrawal and flat affect function as a socially safe way to manage distress without appearing *weak*, supporting the interpretation of anger as a *permitted* masculine channel through which suppressed stress is stored and later released (Courtenay, 2000). It also suggests that suppression disrupts classroom relationships and discipline, where boys may appear compliant in the moment, but the delayed *explosion* destabilizes peer interactions and the wider learning climate. This also shows that overall *learning to switch off* emerges as a form of gendered emotion work in which boys cultivate a *normal face* and emotional distance as a protective performance under intense peer surveillance, aligning with local ideals of being *rough and tough* (Shah & Shah, 2024). Conceptually, this is not an absence of feeling but an embodied regulation project where boys manage visible affect (voice, tears, expression) to prevent vulnerability from being publicly weaponized, making endurance a key marker of respectable manhood (Hochschild, 1979), and in this sense, becoming *mard* through emotional restraint functions as a short-term survival tactic but carries longer-term psychosocial costs, narrowing boys’ emotional repertoires and undermining both learning engagement and supportive peer–teacher ties.

### 3.3. Islands of Care

Against a wider backdrop where emotional exposure can be punished through ridicule, shaming, or being labelled as *weak*, our data also reveal small pockets of relational safety in which boys can momentarily soften the rules of masculine respectability. What we frame as “islands of care” are rarely formal or institutionally guaranteed; they are quiet, situational, and often built through trust, privacy, and tactful communication. Rather than overturning hegemonic masculinity, they make vulnerability conditionally speakable in ways that align with the logic of *caring masculinities* (Way, 2011) while also showing how care remains contingent under peer surveillance and school hierarchies. For instance, one of the participants narrated his views:

*“One newly recruited young teacher is very supportive, with whom I discuss everything… He talks to us privately and tries to understand our problems without teasing or shouting, and if someone says I am feeling stressed, he really supports me. He listens, you know…and tells you what to do next…”*

(Tariq, 15, Private School C, Interview)

Tariq’s account foregrounds two enabling conditions, privacy and non-shaming, which reduce the social risk of disclosure, suggesting that emotional talk becomes possible when the audience is controlled and dignity is protected. This resembles the supportive adult–student relationship in SEL. Still, it emerges less as a standardized program and more as a relational practice anchored in trust and discretion (Brown & Larson, 2009), and it clarifies that care is not necessarily therapeutic or *soft*; it is pragmatic guidance that helps boys navigate stress without jeopardizing status. A second opening toward care appears when teachers reframe failure, not as evidence of incompetence deserving humiliation, but as a normal part of becoming *someone*, and here, care operates as an *emotional pedagogy* that protects boys’ dignity while expanding what can be felt and said in school.

Another participant shared his experiences in this regard, arguing:

*“If you fail a test… some teachers embarrass you in front of everyone…like you’re useless. But one teacher tells us stories… And he says he also failed, other people failed, and still they became something, and then somehow you don’t feel so finished…”*

(Boy 2, 14, Government School B, FGD)

This contrast between public humiliation and story-based reassurance illustrates competing emotional pedagogies that shape what kinds of masculinity are livable in classrooms, and by narrating failure as common and survivable, the teacher offers boys a way to acknowledge disappointment without collapsing into a stigmatized ‘weak’ identity, creating a culturally workable bridge to vulnerability. Importantly, care is not only adult-driven; these boys also described peer-based micro-support, but this openness is carefully calibrated, revealing how care is present yet bounded to manage reputational risk. Farhan narrated his views in this regard:

*“I have a friend or two with whom I discuss a few things happening in our family… but you cannot disclose it with everyone… (..) As they will make fun of you later.”*

(Farhan, 16, Government School A, Interview)

Farhan’s emphasis on *a few things* points to bounded disclosure, where emotional openness is negotiated rather than absent, and the island of care here is relationally contained and restricted to trusted ties and specific topics (e.g., family) that can be framed as legitimate burdens rather than personal weakness. This suggests that peer care is often strategic, as it offers recognition while still respecting masculine feeling rules and the threat of peer surveillance. A school teacher’s account makes the underlying mechanism explicit: boys’ willingness to open up hinges less on *skill deficits* and more on whether emotional talk can occur without being downgraded morally. He argued:

*“They open up when they feel you respect them… and the moment they think you will label them weak or ‘too emotional,’ they close completely. So, I think first you have to show I’m not here to judge you…”*

(Teacher 4, Private School C, Interview)

This perspective identifies respect as emotional currency, shaping disclosure in masculinized environments where vulnerability is easily converted into stigma. This also implies that supportive interventions cannot simply ask boys to *share feelings*; they must actively protect their dignity and social standing to be effective. Taken together, these extracts show that *islands of care* are real but fragile as they appear where privacy, dignity, and trusted relationships interrupt peer surveillance and status threats. They do not erase hegemonic masculinity, and instead, they produce conditional alternatives, forms of caring masculinity that boys can inhabit without losing respect (Elliott, 2016).

### 3.4. Ambivalent SEL Spaces

A clarification is necessary before presenting this theme. None of the three schools was implementing a formally recognized SEL program, and the practices described here (circle discussions, group projects, informal counselling by younger teachers, and occasional pastoral talks) are what we term “SEL-like” activities: school-based routines that engage social–emotional competencies but without structured curricula, training, or fidelity measures. This reflects the reality that in many Global South school contexts, SEL operates not as a standardized programme but as emergent practices embedded in everyday teacher-student interaction (McCoy & Hanno, 2023). Understanding this distinction is important because it was precisely the informal, unstructured character of these practices that made them vulnerable to the gendered dynamics participants described, dynamics in which the same activity could expand empathy under one set of conditions and reproduce stigma under another.

Our data shows that across all three schools, counselling, often facilitated by younger teachers, emerged as the main space where emotional issues were addressed, and participants described practices that look like SEL, including circle discussions, group projects, and occasional informal counselling. Yet, they consistently framed these spaces as double-edged. When teachers actively protected students from mockery and framed emotions as ordinary and manageable, these activities could expand empathy and reflective talk; still, when disclosures were met with laughter, or when adults implicitly gendered emotions as *for girls*, the same activities became sites of re-stigmatization, reproducing the very shame they were meant to reduce. In line with critical work on schooling and affect, these accounts underscore that SEL is never purely technical; it is enacted within existing hierarchies of gender and status and can therefore either expand boys’ emotional possibilities or reinscribe the *boys don’t cry* rules (Evans, 2017; Jagers et al., 2025).

One student’s account makes the basic mechanism visible, where he pointed out that the moment vulnerability is publicly ridiculed, *sharing* becomes a liability, and the space collapses. He further argued:

*They tell us, ‘share your feelings,’ like it’s a good thing… but if someone says something real… like something serious… boys start laughing and making comments. And who would want to speak again, because you know it will come back to you…*

(Hasan, 13, Government School A, FGD)

A second account from a student highlights that even when SEL-like activities build cooperation, emotional expression can still trigger gender policing, limiting which competencies boys can safely perform. He argued:

*In group projects, you learn to work together… like who does what, how to help each other. But if a boy gets emotional or looks upset, others immediately say, why are you acting like a girl…?’ Then he tries to hide it and becomes quiet.*

(Kashif, 15, Government School B, Interview)

Kashif’s account captures the conditional nature of SEL competencies, in which collaboration is acceptable but visible vulnerability is sanctioned through feminizing insults, illustrating how relationship skills and self-awareness, core aims of SEL, are constrained by hegemonic masculinity’s disciplinary boundary work (CASEL, 2020; R. W. Connell & Messerschmidt, 2005). The outcome is not simply a *lack of emotional skills*, but socially induced concealment, in which boys learn that cooperation is safe while emotional openness carries status penalties.

Teachers, in turn, described facilitation not as a neutral *activity*, but as ongoing governance of interaction, and without that governance, the circle format can intensify the power of the peer audience. One teacher narrated his views:

*Circle talk only works if I control the jokes and if I don’t stop it quickly, it turns into teasing and then it becomes bullying. So the first job is not the circle, but the first job is discipline and respect.*

(Teacher 6, Government School A, Interview)

This teacher reframes SEL as boundary-setting where emotional learning requires active regulation of what counts as acceptable peer interaction, not just an invitation to share, supporting the argument that SEL’s outcomes hinge on protective mechanisms with clear rules, immediate intervention, and respect-enforcing practices, without which *participation* can magnify humiliation (Zieher et al., 2024), or in other words, the pedagogy of SEL is inseparable from the politics of the classroom.

Finally, a student’s critique exposes an institutional contradiction in which SEL messages can be undercut when teachers simultaneously reproduce hegemonic masculinity through moral talk. He argued:

*Sometimes in the lesson the teacher would say, ‘anyone can talk about emotions, don’t keep it inside.’ But then the same teacher will say, ‘Men are strong, they don’t cry.’ So what is the point? If crying is shameful… then their lessons are just words.*

(Arif, 16, Private School C, Interview)

Arif identifies the double bind produced when schools promote emotional literacy while reaffirming that *real men don’t cry*, turning SEL into a space of mixed signals and mistrust. Analytically, this shows SEL as contested terrain where competing moral orders coexist where one invites reflection, the other restores gendered feeling rules (Hochschild, 1979), and the implication is that SEL’s effects depend on whether educators interrupt or reproduce masculine scripts, without alignment at the level of everyday teacher talk, SEL risks reinforcing shame rather than reducing it (Durlak et al., 2011).

## 4. Discussion

Our findings underline that boys’ emotional lives in Pakistani secondary schools are best understood not as an individual *lack of skills*, but as a social accomplishment shaped by gendered status relations, and across the three school sites, boys’ accounts show that emotions are evaluated publicly through peers, discipline, and reputational logics so that feeling and display become matters of masculine legitimacy. This supports a core claim of critical masculinity studies that masculinities are relational, hierarchical, and institutionally produced rather than private traits (R. W. Connell & Messerschmidt, 2005). It also extends sociologies of emotion by illustrating how gendered *feeling rules* operate in adolescence as practical constraints on what boys can safely show, and how boys engage in sustained emotion work to remain intelligible as *proper* men (Hochschild, 1979, 1983).

The data suggest that sadness, fear, and crying are widely constructed as high-risk signals that can trigger ridicule and loss of standing, while anger and toughness are treated as legitimate, even protective, masculine performances, results that align with scholarship showing how hegemonic masculinity is maintained through the stigmatization of vulnerability and the valorization of control (R. W. Connell & Messerschmidt, 2005). Conceptually, boys’ narratives clarify a mechanism often assumed rather than demonstrated, where distress is not absent but is frequently translated into an affective register that *fits* masculinity, most notably anger, because anger can preserve respect, deter teasing, and restore status. This speaks directly to work on emotional pedagogy and the gendering of emotional expression in school contexts, where institutional routines and peer cultures teach students what emotions count as appropriate for boys (Evans, 2017), making emotional exposure feel materially unsafe rather than merely culturally discouraged.

The descriptions of *switching off* by our participants deepen this picture by showing that emotional suppression is an active, learned practice, and many boys depict numbing as a marker of becoming *mard* (a disciplined masculinity characterized by endurance and self-control). Yet, they also link it to loneliness, irritability, and reduced engagement with learning. This is analytically important because it reframes emotional silence as gendered emotion work, where these boys are not simply failing to name feelings; they are strategically managing feelings and displays to avoid sanctions (Hochschild, 1979). Where the social costs of vulnerability are high, *not feeling* (or performing not feeling) becomes a rational solution, even if it has longer-term psychosocial and educational costs. This finding complicates skills-based SEL framings that treat emotion regulation as universally desirable and culturally neutral, because in these schools, regulation is already happening, and often through suppression shaped by masculine feeling rules.

Importantly, not all accounts of emotional restraint in our data can be read solely through a deficit lens. Some boys described managing emotions in terms that evoke the Pashtun moral concept of *sabar* (patience/endurance), a culturally sanctioned capacity to endure difficulty without public complaint, valued across Islamic and Pashtun ethical traditions (Ahmed, 2013). Where boys framed self-control as a skill, a source of dignity, or a way of protecting family honour, their accounts point to the functional and morally meaningful dimensions of emotional regulation that coexist with, but are not reducible to, the costs of gendered suppression. The critical distinction our data illuminate is contextual: emotional restraint becomes problematic not when boys choose composure as a culturally meaningful practice, but when it is socially coerced under peer surveillance and institutional punishment, leaving boys with no relational outlet for distress. This dual reading, attending to both the cultural value and the social costs of emotional restraint, avoids imposing a blanket deficit interpretation while still identifying the conditions under which *becoming mard* through endurance narrows emotional repertoires and undermines well-being.

At the same time, our findings resist any deterministic conclusion that boys are uniformly committed to emotional toughness, and accounts of *islands of care* show that alternative emotional practices are already present, though unevenly distributed and fragile. When particular teachers, counsellors, or trusted peers offered privacy, dignity, and non-shaming responses, boys described being able to acknowledge worry, failure, or family stress without immediate status loss. These moments resonate with the concept of caring masculinities, which captures emergent masculine practices oriented toward relationality, emotional openness, and care rather than domination (Elliott, 2016). Importantly, our data suggest that caring masculinities in this context do not typically appear as public performances, instead they are often situational, backstage, and mediated through culturally workable forms such as humor, storytelling, or informal mentoring, extending caring masculinities scholarship by showing how care can become viable even under strong *boys don’t cry* norms, but primarily when the interactional conditions reduce the risk of feminization and ridicule.

Although the study was not designed as a formal comparative analysis, some cross-site patterns merit noting. Boys in government schools, where corporal punishment and larger class sizes were more commonly reported, described emotional suppression as more tightly linked to fear of teacher-enacted violence and public humiliation (Khan et al., 2025), whereas boys in the private school more frequently referenced peer-level status competition and parental academic pressure as drivers of emotional concealment. Similarly, “islands of care” emerged more readily in the private school, where a younger teacher with counselling training was available, while government school participants described care as episodic and dependent on individual teacher initiative rather than institutional structures, a pattern consistent with research showing that school-based psychosocial support in Pakistan remains unevenly distributed and under-resourced (Dayani et al., 2024; Hamdani et al., 2021). These patterns suggest that institutional resources and disciplinary climate shape the specific mechanisms through which masculinity norms operate in school emotional life, even if the overarching logic of gendered feeling rules was consistent across sites (Hochschild, 1979; Evans, 2017). This institutional variation has direct implications for how SEL-like practices function across different school contexts.

These cross-site differences underscore the broader point where our study makes its strongest intervention into SEL scholarship, showing that SEL-like practices (circle discussions, group work) can open space for empathy and reflection but can also reproduce stigma if vulnerability is framed as unmanly or if disclosure is left unprotected from peer mockery (Shah et al., 2022; Shah, 2026).

Our findings therefore provide empirical grounding for transformative SEL, which argues that SEL must engage power and social identities, rather than assuming a universal learner who can safely disclose and practice emotional skills in any classroom (Lin et al., 2023), which in other words, our data show that whether SEL *works* depends not only on activities, but on the social meaning of those activities within local masculinity regimes. However, this does not diminish SEL’s promise; it clarifies the conditions under which SEL is likely to be effective for boys in high-stigma settings, and the broader evidence base indicates that well-implemented universal school-based SEL programs improve social–emotional competencies and a range of behavioural and academic outcomes (Durlak et al., 2011; Taylor et al., 2017). Our contribution is to specify what *good implementation* must include when masculinity norms shape emotional risk, with explicit norm-setting against ridicule, facilitation strategies that protect dignity, and pedagogies that do not treat sadness or fear as incompatible with respect. The accounts also suggest pragmatic entry points, such as beginning with emotions boys can name without immediate status loss (stress, anger) and gradually expanding emotional repertoires once psychological safety is built, without letting anger remain the only legitimate masculine affect. This is a concrete way in which our study extends both masculinity theory and SEL practice, as it conceptualizes schools as sites where boys’ emotional repertoires are negotiated under constraint, and it positions SEL as an arena of gender politics rather than as neutral skills training. Concretely, our data point to five facilitation conditions that shaped whether SEL-like practices expanded or narrowed boys’ emotional repertoires.

Drawing on participants’ accounts, we propose five findings-grounded design principles for gender-attentive SEL in high-stigma contexts. First, begin with “safe” emotions and expand gradually, as our data showed, boys engaged more readily with stress and frustration than with stigmatized affects like sadness or crying, suggesting that starting with less feminized emotions builds emotional vocabulary while reducing reputational risk. Second, establish and enforce anti-ridicule norms before disclosure, as teachers consistently reported that SEL activities collapsed when mockery went unchecked, explicit ground rules and immediate intervention were prerequisites, not afterthoughts. Third, protect privacy and use indirect emotional pedagogy, boys opened up more in one-on-one settings than peer-facing groups, and indirect entry points (storytelling, hypothetical scenarios) reduced the personal exposure that triggers gender policing. Fourth, normalize failure through teacher self-disclosure, participants described teachers who shared their own struggles as among the most powerful “islands of care,” reframing vulnerability as universal rather than as evidence of weakness. Fifth, align SEL messaging with teachers’ everyday gender talk, participants identified a corrosive double bind when teachers promoted emotional openness but simultaneously reinforced “real men don’t cry” scripts, indicating that gender-attentive SEL requires not only specific activities but institutional coherence in how educators talk about masculinity across all interactions.

Beyond these practical implications, our study contributes empirically by centering boys’ voices from an underrepresented Global South context and by linking emotion talk to the lived realities of school discipline, peer surveillance, and respectability. At the same time, theoretically, it integrates hegemonic masculinity with emotion-work approaches to show how boys’ emotional suppression is socially produced and repeatedly reinforced in school life (R. W. Connell & Messerschmidt, 2005; Hochschild, 1979, 1983). It also advances caring masculinities by demonstrating how *care* becomes possible in small, relational pockets rather than as wholesale norm change (Elliott, 2016; Shah, 2026). For SEL research, the study provides a context-specific account of why gender neutrality is not merely an omission but a risk, and it can turn SEL into an exposure event for boys if vulnerability is invited without protection and without challenging *unmanly emotions* scripts (Jagers et al., 2025).

Finally, these findings should be interpreted with consideration of their limitations. The study is qualitative and situated in three urban schools in Khyber Pakhtunkhwa, and patterns may differ in rural areas, other provinces, elite private schools, madrasa contexts, or among out-of-school boys. While our sample spanned ages 13–16, the study was not designed to examine developmental differences; tentatively, younger participants (13–14) described emotional concealment as reactive, learned through specific incidents, while older participants (15–16) framed restraint as a settled aspect of masculine identity, consistent with research on the internalization of suppression across adolescence (Zimmermann & Iwanski, 2014), but requiring longitudinal designs to confirm. Additionally, although class differences were apparent in participants’ accounts, with government school boys more frequently referencing economic insecurity and household labour as compounding emotional pressures, our data do not permit systematic analysis of how socioeconomic position intersects with masculinity and emotion; future research could benefit from deliberate socioeconomic stratification and from integrating economic precarity as an analytic category alongside gender. Even with these limits, our analysis demonstrates a central point: Boys don’t cry is not simply an attitude within these schools; it is a socially enforced rule that shapes how distress becomes visible, speakable, and governable.

## 5. Conclusions

In conclusion, this study shows that Pakistani schoolboys’ emotional lives are shaped less by an absence of feelings than by gendered feeling rules that make sadness, fear, and crying socially risky, while rendering anger and toughness legitimate ways to protect masculine status. Within these school ecologies, boys learn to *switch off* emotions as a form of masculinized emotion work that can reduce immediate ridicule yet carries costs for well-being, relationships, and learning engagement, while at the same time, the presence of *islands of care* demonstrates that boys can and do value relational support and emotionally open masculinities when dignity is protected, pointing toward the practical relevance of caring masculinities as an emergent possibility in high-stigma contexts (Elliott, 2016). Finally the study shows that SEL-like practices can either open up or close down space for non-violent, emotionally expressive masculinities where they foster empathy and reflection when classrooms are made psychologically safe and vulnerability is treated as compatible with respect, but they reproduce stigma when emotional disclosure is exposed to mockery or framed as “unmanly,” underscoring the need for gender-attentive, transformative SEL approaches in Pakistani secondary schools.

## Data Availability

The data that support the findings of this study are available from the corresponding author, upon reasonable request. Due to the sensitive nature of the interviews with students and school staff, data are not publicly available to protect participant privacy.

## Abbreviations

The following abbreviations are used in this manuscript:

UNICEF	United Nations International Children’s Emergency Fund
WHO	World Health Organization
UNESCO	United Nations Educational, Scientific and Cultural Organization
UNHCR	United Nations High Commissioner for Refugees
CASEL	Collaborative for Academic, Social, and Emotional Learning
SEL	Social and Emotional Learning
FGDs	Focus Group Discussions
